# Recurrence of a dermatofibrosarcoma protuberans? Rare case

**DOI:** 10.1016/j.radcr.2024.06.096

**Published:** 2024-08-05

**Authors:** Sohayb Darraz, Zakaria khattab, Ilyesse Haichour, Omar Mokhtari, Amine El Farhaoui, Adnane Lachkar, Najib abdeljaouad, Hicham Yacoubi

**Affiliations:** a Faculty of Medicine and Pharmacy, Mohammed I University, Oujda, Morocco; b Department of Traumatology, orthopedic Mohammed VI University Hospital Mohammed I University, Oujda, Morocco

**Keywords:** Dermatofibrosarcoma protuberans, CD34-positive tumor, Skin cancer

## Abstract

Dermatofibrosarcoma protuberans (DFS) is a mesenchymal-origin skin tumor with intermediate malignancy. Though rare, it's not exceptional, comprising about 0.1% of malignant skin tumors.

The authors discuss clinical, radiological, histopathological studies, and various therapeutic modalities for this tumor.

Our 82-year-old patient presented with a 3 cm swelling on the right arm, initially undergoing biopsy followed by surgical excision of the mass.

Adjuvant treatment with radiotherapy or chemotherapy is unnecessary unless recurrence or malignant transformation occurs. Histological analysis is crucial for diagnosis. The preferred treatment method is wide surgical excision.

Prognosis primarily depends on malignancy, especially at the local level, with a high risk of recurrence. It's rare for a distinctly malignant sarcomatous transformation with metastasis to occur.

## Introduction

Dermatofibrosarcoma protuberans (DFS) constitutes a rare but nonatypical form of malignant mesenchymal skin tumor. It represents approximately 0.1% of all malignant skin tumors and accounts for less than 5% of all soft tissue sarcomas in adults [[1], [2], [3]].

It is a tumor formation positioned between the spectrum of benignity, characterized by the very common and harmless cutaneous fibroma, and that of malignancy, represented by the true cutaneous fibrosarcoma. Its transition to a distinctly malignant nature with metastases is remarkably rare [2].

The trunk is the preferred site, followed by proximal extremities, and then the head and neck [3].

This tumor, whose incidence is notable in African nations [4], poses several challenges due to various factors: its lack of recognition by most general practitioners and even some specialists, its deceptive clinical appearance which can often be confused with a keloid scar, leading to frequent diagnostic delays, its severity attributed to its local aggressiveness and destructive potential, as well as its recurrent nature in cases of noncompliance with the rigorous protocols required for managing this specific tumor.

## Case presentation

This is an 82-year-old patient with a history of Type 2 Diabetes treated with oral antidiabetic medication and hypertension managed with monotherapy, pacemaker 20 years ago.

The patient's history dates back 40 years with the appearance of a mass on the right shoulder, which reportedly underwent tumor resection. The patient experienced good recovery with no signs of recurrence or other complications. However, the mass reappeared at the same location 2 months ago, with a progressive increase in size, but no associated general symptoms such as weight loss or loss of appetite.

Physical examination reveals a patient in good general condition, with a localized mass in the upper third of the right arm measuring 3 cm in the longest axis. The mass is soft, fixed superficially, and mobile deep down, without signs of inflammation.

**Fig. 1:**
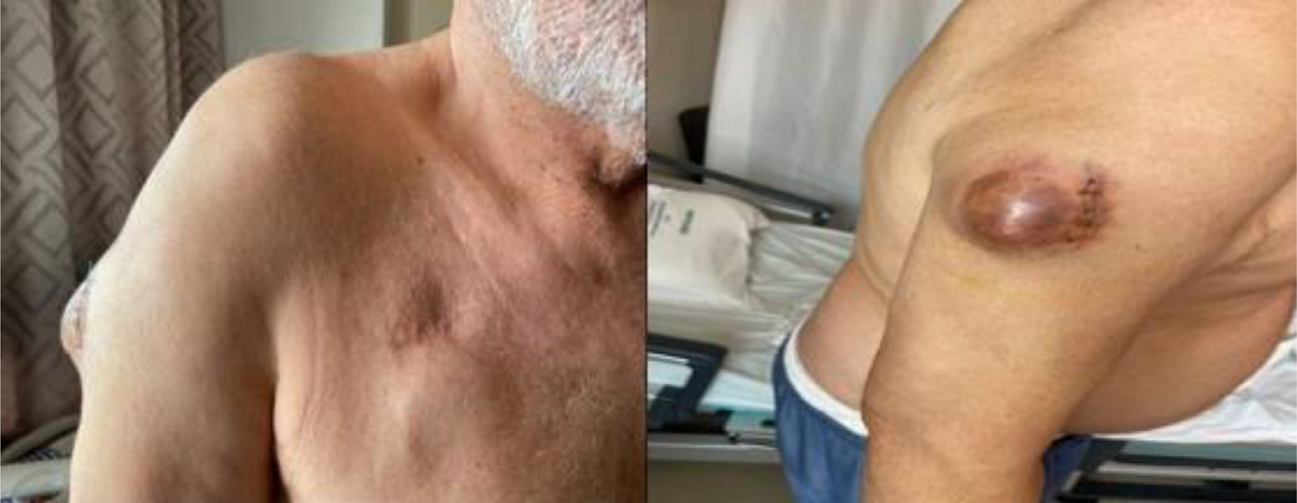
Showing the clinical species of the mass.

It has a solid consistency (Fig. 1), is painless on palpation, and does not affect functionality. There are no palpable lymph nodes or other concerning signs.

In this recurrent case, performing an MRI is crucial. However, since the patient has an absolute contraindication to MRI due to a metallic implant, a CT scan of the tumor was conducted instead. MRI is typically the preferred imaging technique for its superior soft tissue contrast and detailed imaging capabilities. Yet, the presence of metallic implants can cause significant artifacts and pose safety risks, making MRI impractical or dangerous.

Consequently, a CT scan, which provides adequate imaging without the interference of metallic objects, was chosen. CT imaging is effective in visualizing the tumor and assessing its characteristics, ensuring proper diagnostic evaluation despite the MRI contraindication.

**Fig. 2:**
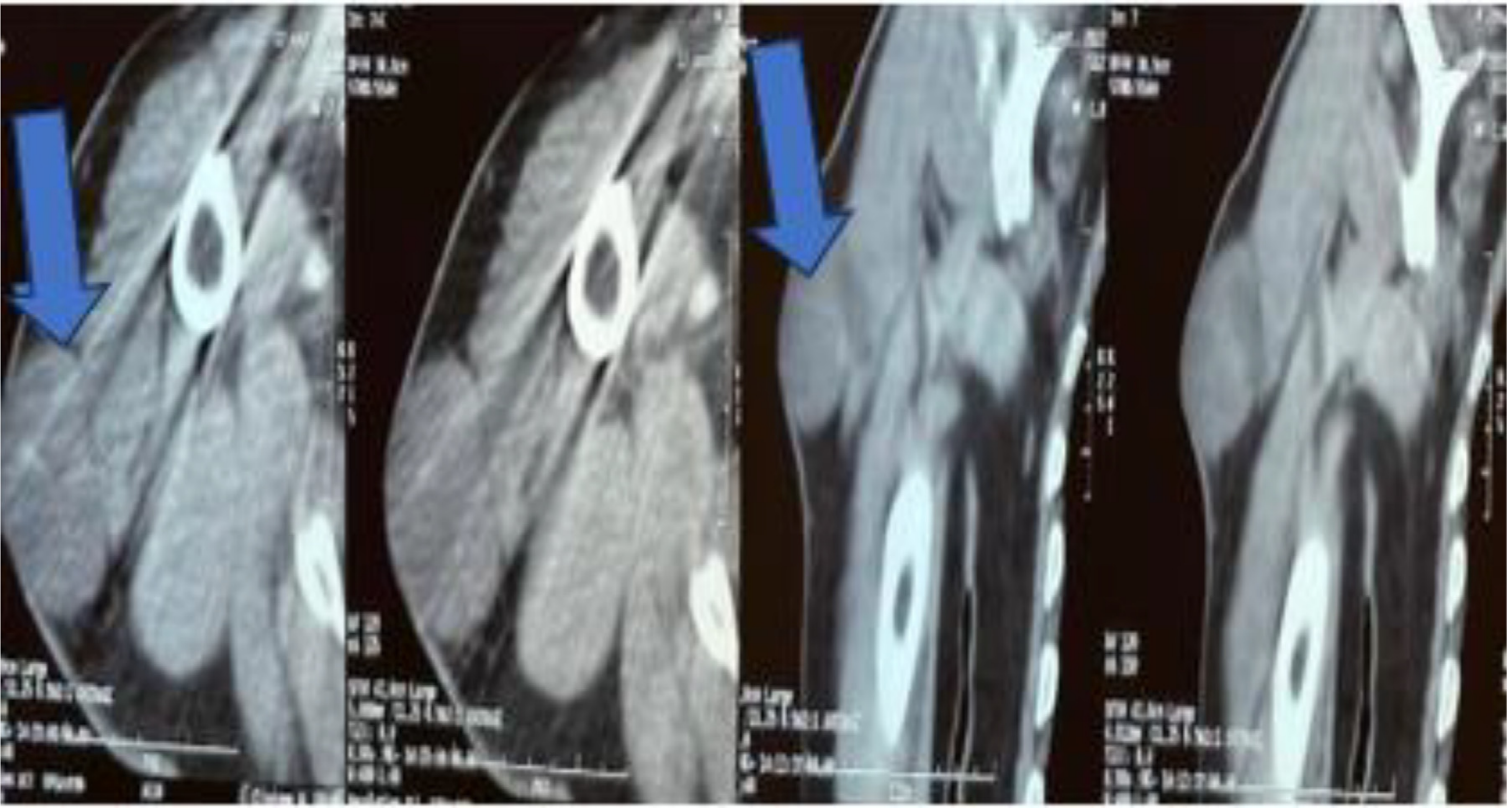
Showing CT scan of the arm revealing a subcutaneous mass in the upper third of the outer aspect of the right arm, well-defined oval in shape, with regular contours, heterodense on contrast, containing a large liquid area and a fleshy component enhanced after contrast. This mass infiltrates the skin covering and presents intimate contact with the deltoid muscle with persistence of a fatty separation border.

Radiological exploration with a CT scan (Fig. 2) of the right arm and a biopsy were performed, confirming a diagnosis of dermatofibrosarcoma protuberans.

**Fig. 3:**
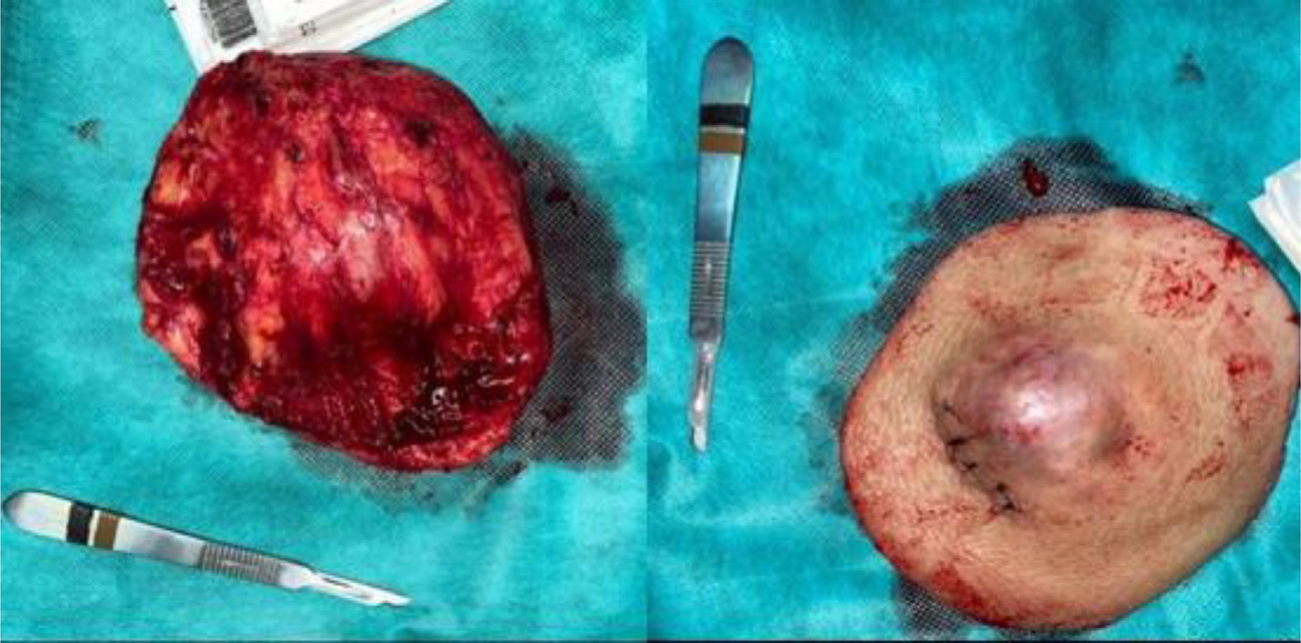
The macroscopic appearance of the operative specimen.

Subsequently, the patient was taken to the operating room for a potential tumor resection. For this reason, he was admitted to the operating room in 2 stages. In the first stage, under general anesthesia, a resection with a 5cm safety margin was performed. The entire mass was detached as a single unit, including the deep fascia without tumor breach, ensuring a macroscopically clear resection margin. The surgical specimen was then sent for pathological examination (Fig. 3).

**Fig. 4:**
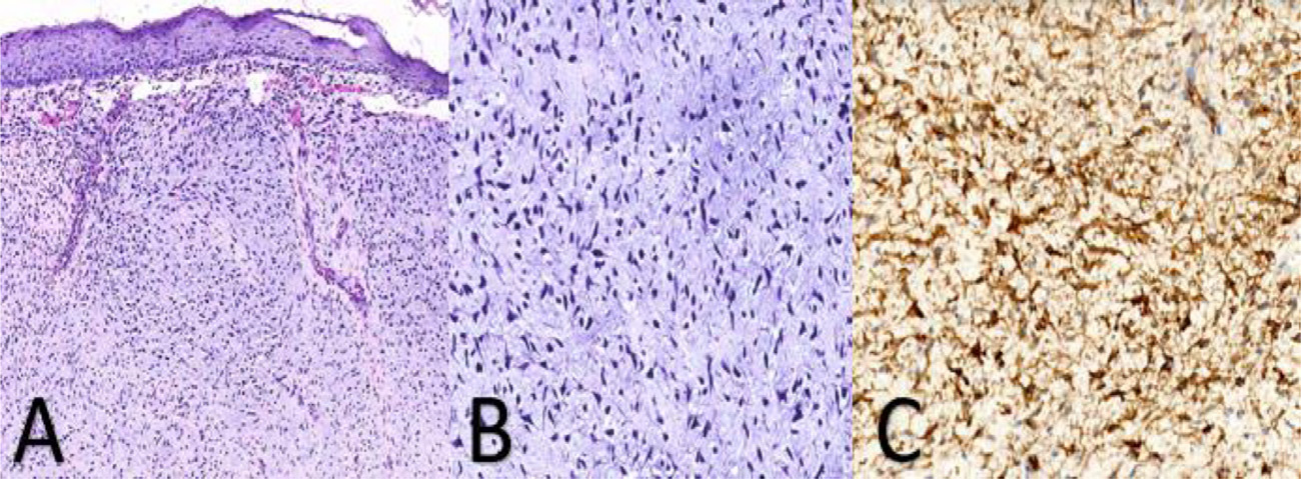
Microscopically, the resection reveals a cutaneous tissue with dermis largely infiltrated by a proliferation arranged in diffuse sheets, with a myxoid appearance stroma (A: HE, x200). At high magnification, the tumor cells are spindle-shaped, with hyperchromatic, irregular nuclei and scant, eosinophilic cytoplasm (B: HE, x400). The tumor cells exhibit intense and diffuse staining with CD34 (C).

The pathological examination confirmed a dermatofibrosarcoma of Darier and Ferrand with myxoid differentiation (Fig. 4).

**Fig. 5:**
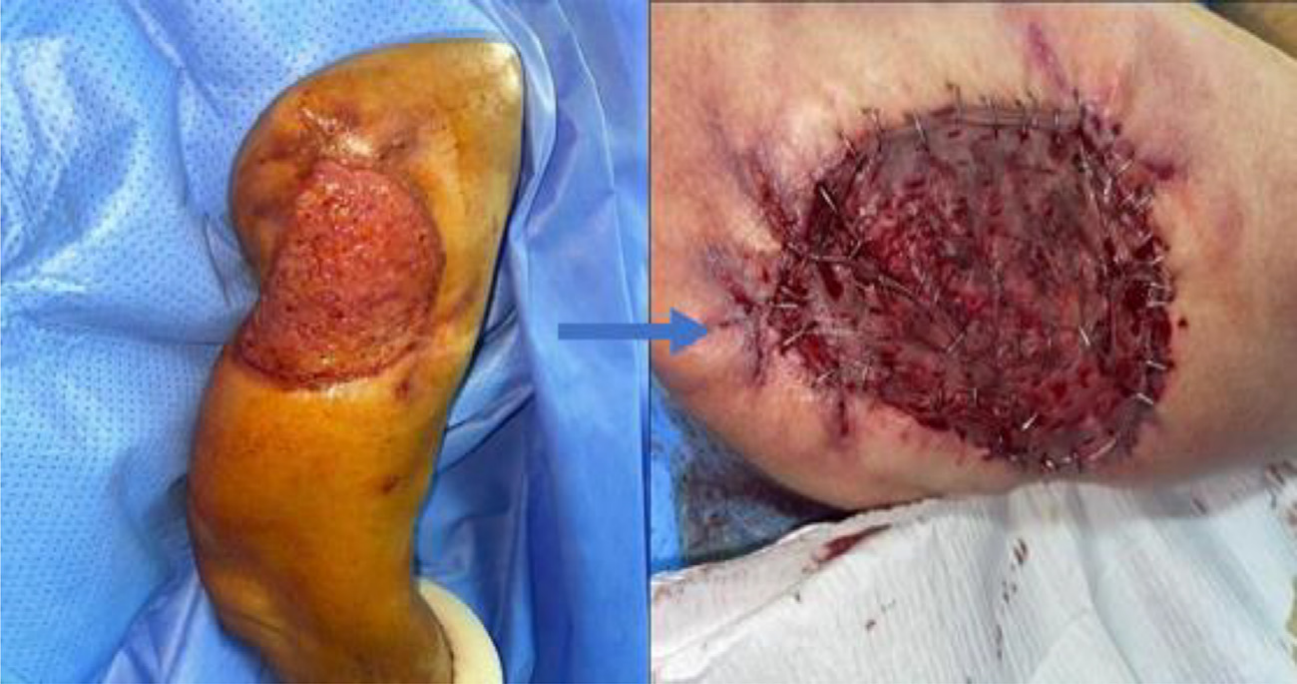
Skin appearance before and after skin grafting.

In the second stage, after 6 weeks, a skin graft was performed using skin from the inner thigh to fill the skin defect, estimated to be 8 cm in length and 5 cm in width (Fig. 5).

The progression was marked by successful skin healing without signs of local or regional recurrence.

**Fig. 6:**
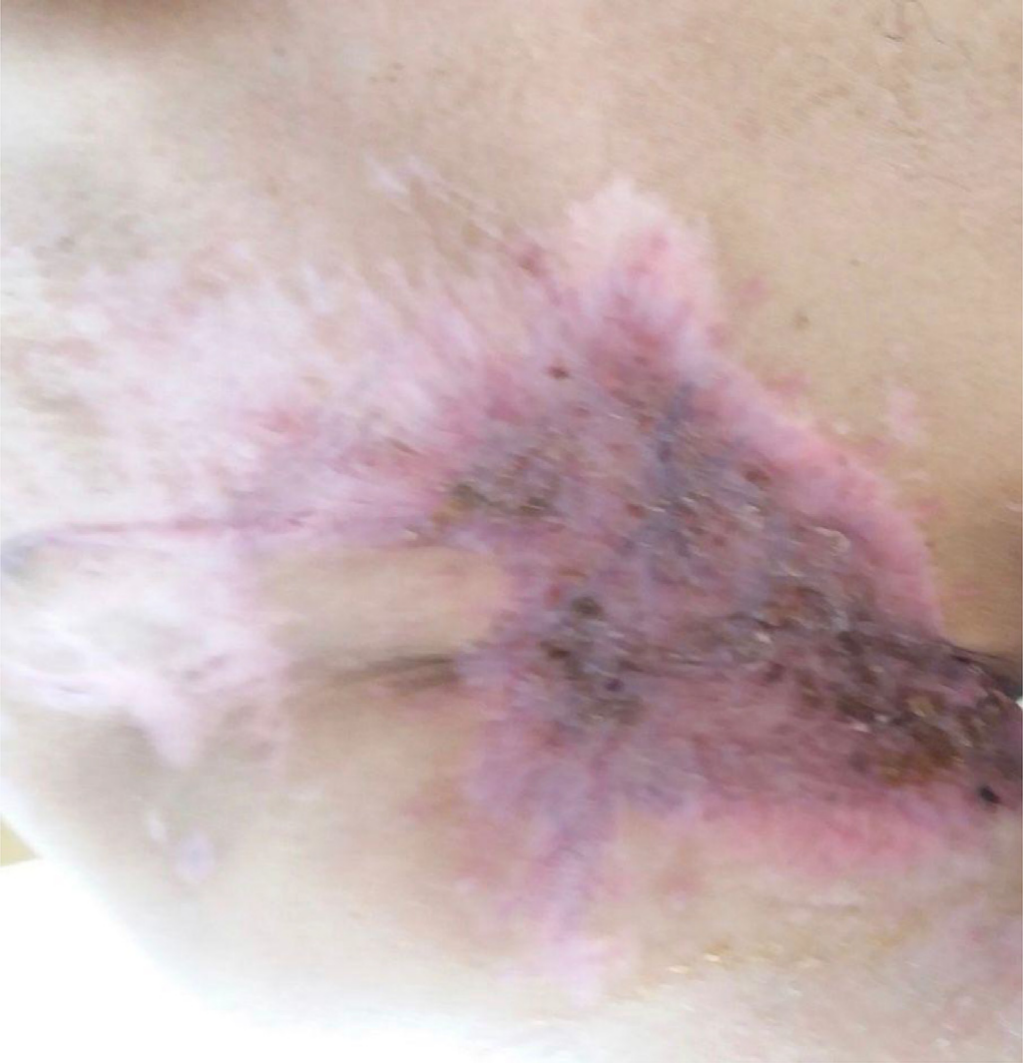
Clinical image after 2 years showing the scar and absence of recurrence.

In our case, after preanesthetic preparation of the patient, we opted for a surgical treatment involving tumor resection with a 5 cm safety margin and superficial fascia resection. The operative specimen was sent for histopathological examination. Neoadjuvant radiotherapy was not administered as the tumor was easily accessible and resectable, and chemotherapy was not given as the patient did not present with metastases. The patient was monitored regularly: every 5 days during the first 2 months following the graft placement, then monthly in the first year, and every 2 months in the second year (Fig. 6). The patient showed good progress and healing with no evidence of local recurrence or new mass formation.

## Discussion

Among all available definitions, the one proposed by DEGOS [5] appears to be the most comprehensive: “It is a dermal connective tissue tumor with spindle cells, presenting a histological structure more or less similar to that of sarcomatous tumors. However, it is distinguished from truly primitive fibrosarcomas by its exclusively cutaneous origin and its extremely slow evolution. At a very advanced stage, a frankly malignant sarcomatous transformation with metastatic potential occurs only exceptionally.”

It constitutes 1% of all soft tissue sarcomas, with a prevalence ranging between 0.8 and 5 cases per million inhabitants each year. Disease-free survival (DFS) rates are particularly high in individuals of African origin, with an equal male-to-female ratio (1:1) and a 5-year relative survival rate of 99% [6,7].

Generally, the diagnosis is made in individuals aged 20 to 59 years, although congenital and pediatric cases have also been reported [8].

DFS can affect any region of the body. According to information available in the literature, there is a notable preference for the trunk, affected in 50% to 60% of cases. The extremities represent between 20% and 30% of locations, while the head and neck are involved in 15% to 20% of cases [[9], [10], [11]].

In a study encompassing 115 cases, Taylor and Helwig identified a history of trauma in 16.5% of observations [11].

Regarding clinical manifestations, initially, the lesion presents as an indurated plaque covered by skin with a normal appearance, sometimes whitish, white-yellowish, pink, purplish, or reddish.

It appears well-defined and mobile compared to deeper planes. At a later stage, the plaque expands, its surface becomes irregular and lumpy, evolving over several months to several years into a multinodular mass, often polychromatic, of variable size, hard, and perfectly mobile on deeper planes. However, this 2-stage evolution is not systematic, as some forms initially present with single or multinodular nodules with subsequent fusion of the nodules. Cases of “monstrous tumors” reaching up to 6.5 or even 7 kg have been documented.

These tumors can reach considerable dimensions, up to 25 cm in diameter, and the lesion can develop on any part of the body [12].

Ninety percent of DFSPs are low-grade lesions, while sarcomatous transformation is observed in 10% to 15% of cases, usually in the form of low-grade fibrosarcoma. This histological distinction has been associated with increased tumor aggressiveness. The fibrosarcomatous subtype of DFSP is an unfavorable predictive factor for disease-free survival. Therefore, this subtype is considered to have a higher rate of local recurrence and metastasis [7].

CD34 is typically positive in DFSP (positive in 92% to 100% of cases), while factor XIII-a is negative, with an inverse staining pattern found in FSDF [13]. Some cases of FSDF show positive staining for CD34; however, staining areas are less diffuse and usually observed at the periphery of the neoplasm. CD34 expression is often reduced or lost in DFSP with fibrosarcomatous transformation [14].

Mori et al. found in 2008 that nestin was expressed in 94% of DFSP cases [15].

PDGFB protein expression is also a possible histological marker for DFSP .

The complexity of treatment arises from the subclinical extension of the tumor, which can lead to recurrences. Thus, wide excision surgery remains the standard treatment, requiring safety margins of 4 to 5 cm and removal of the superficial fascia [[16], [17], [18]] . Some advocate for postoperative radiotherapy from the second recurrence [18], while systemic chemotherapy is generally not recommended [19].

In advanced or metastatic DFS cases, targeted therapy is employed. Imatinib mesylate (STI571) is a major targeted therapy for DFSP. The role of imatinib in managing these conditions is derived directly from its mechanism of action: it is a tyrosine kinase inhibitor targeting BCR/ABL (used in chronic myeloid leukemia), KIT (used in gastrointestinal stromal tumors), FMS (receptor for colony-stimulating factor 1), and PDGFR alpha and beta (used in DFSP) [20].

Close clinical surveillance is imperative due to the slow evolution and high potential for recurrence of this tumor [18].

Frankly malignant metastasizing sarcomatous transformation is rare and occurs at a very advanced stage [12]. Its pronounced potential for recurrence, even after often extensive surgical excisions, confers upon this lesion a clinically challenging nature [19].

Previously reported local recurrence rates for DFSP range from 20% to 55%. Most local recurrences occur within the first 3 years postresection [[3], [21], [22]]. However, about 30% of these recurrences are observed after 5 years [23]. This underscores the need for prolonged follow-up in patients to promptly identify and manage late recurrences.

## Conclusions

Dermatofibrosarcoma Darier-Ferrand represents a rare fibrous skin tumor, characterized by its diagnostic difficulty, very slow local progression, and propensity for local recurrence, with rare metastases. Due to its high recurrence potential, regular clinical monitoring is necessary. Both diagnostic and therapeutic challenges require precise confirmation of the diagnosis through histological analysis, supported by immunohistochemical study. The examined series demonstrates clinical, histological, and evolutionary similarities in accordance with the literature data.

## Patient consent

Written informed consent was obtained from the patient for publication of this case report and accompanying images. A copy of the written consent is available for review by the Editor-in-Chief of this journal on request.

## References

[1] Monnier D., Algros M.P., Vidal M.C., Danzon A., Pelletier F., Puzenat E. (2005).

[2] Joucdar S, Kismoune H, Boudjemia F, Acha D, Abed L. (2001). Les dermatofibrosarcomes de Darier et Ferrand: analyse r étrospective de 81 cas sur dix ans (1983-1994). Ann Chir Plast Esthét.

[3] Stojadinovic A, Karpoff HM, Antonescu CR, Shah JP, Singh B, Spiro RH (2000). Dermatofibrosarcoma protuberans of the head and neck. Ann Surg Oncol.

[4] Kneebone Rl, Melissas J, Mannell A. (1984). Dermatofibrosarcoma protuberans in black patients. S Afr Med J.

[5] Degos H, Civatte J, Belaich S. Dermatofibrosarcome de DarierFerrand. (Dermatofibrosarcome protubérant d'HOFFMANN). Dermatologie – Edition Flammarion Paris, 1981, tome II: 875–877

[6] Rouhani P, Fletcher CD, Devesa SS, Toro JR. (2008). Cutaneous soft tissue sarcoma incidence patterns in the U.S. : an analysis of 12,114 cases. Cancer.

[7] Harati K, Lange K, Goertz O, Lahmer A, Kapalschinski N, Stricker I (2017). A single-institutional review of 68 patients with dermatofibrosarcoma protuberans: wide re-excision after inadequate previous surgery results in a high rate of local control. World J Surg Oncol.

[8] Saiag P, Grob JJ, Lebbe C, Malvehy J, del Marmol V, Pehamberger H (2015). Diagnosis and treatment of dermatofibrosarcoma protuberans. European consensus-based interdisciplinary guideline. Eur J Cancer.

[9] Bendix-Hansen K, Myhre-Jensen O, Kaae S. (1983). Dermatofibrosarcoma protuberans: a clinicopathological study of nineteen cases and review of the world literature. Scand J Plast Reconstr Surg.

[10] Burkhardt BR, Soule EH, Winkelmann RK, Ivins JC. (1996). Dermatofibrosarcoma protuberans: study of fifty-six cases. Am J Surg.

[11] Taylor HB, Helwig EB. (1962). Dermatofibrosarcoma protuberans: a study of 115 cases. Cancer.

[12] Kasse A, Dieng M, Deme A, Fall MC, Drabo B, Timbely G (1999). Les dermatofibrosarcomes de darier et ferrand, à propos de 22 cas et revue de la littérature. Médecine d'Afrique Noire.

[13] Luu C, Messina JL, Brohl AS, Raghavan D, Ahluwalia MS, Blanke CD (2017). Textbook of uncommon cancer.

[14] Serra-Guillén C, Llombart B, Nagore E, Requena C, Traves V, Llorca D (2013). High immunohistochemical nestin expression is associated with greater depth of infiltration in dermatofibrosarcoma protuberans: a study of 71 cases. J Cutan Pathol.

[15] Mori T, Misago N, Yamamoto O, Toda S, Narisawa Y. (2008). Expression of nestin in dermatofibrosarcoma protuberans in comparison to dermatofibroma. J Dermatol.

[16] Morel M, Taïeb S, Penel N, Mortier L, Vanseymortier L, Robin YM (2011). Imaging of the most frequent superficial soft-tissue sarcomas. Skeletal Radiol.

[17] Bianchi L, Maire G, Pedeutour F. (2007). De la cytogénétique à la cytogénomique du dermatofibrosarcome de Darier-Ferrand (dermatofibrosarcoma protuberans) et des tumeurs apparentées. Bull Cancer.

[18] Boujelbenea N, Elloumia F, Hassinea SB, Frikhab M, Daouda J. (2009). Le dermatofibrosarcome de Darier et Ferrand: à propos de 11 cas. Cancer/Radiothérapie.

[19] Nedelcu I., Costache D.O., Costache R.S., Nedelcu D., Nedelcu L.E. (2006). Darier-Ferrand Dermatofibrosarcoma protuberans with peculiar aspect. BMMR.

[20] Pagès C., Kérob D., Lebbé C. (2013). Thérapies ciblées et dermatofibrosarcome de Darier et Ferrand. Oncologie.

[21] Simstein NL, Tuthill RJ, Sperber EE, Kovalcik PJ, Mullen JT. (1977). Dermatofibrosarcoma protuberans—case reports and review of literature. South Med J.

[22] Batsakis JG, Manning JT. (1986). Soft tissue tumors: unusual forms. Otolaryngol Clin N Am.

[23] Rutkowski P, Debiec-Rychter ˛ M, Nowecki Z, Michej W, Symonides M, Ptaszynski K (2011). Treatment of advanced dermatofibrosarcoma protuberans with imatinib mesylate with or without surgical resection. J Eur Acad Dermatol Venereol.

